# Atorvastatin as an adjuvant with betamethasone valerate reduces disease severity and cardiovascular risks in Psoriasis

**DOI:** 10.12669/pjms.336.14068

**Published:** 2017

**Authors:** Farah Asad, Moosa Khan, Fatima Rizvi

**Affiliations:** 1 Dr. Farah Asad, MBBS, MPhil, Associate Professor, Department of Pharmacology, Jinnah Medical and Dental College, Shaheed-Millat Road, Bhaderabad, Karachi, Pakistan; 2 Dr. Moosa Khan, MBBS, MPhil, PhD. Head and Professor, Department of Pharmacology, Shaheed Zulfiqar Ali Bhuttoo Medical University, Islamabad, Pakistan.Department of Pharmacology, Jinnah Medical and Dental College, Shaheed-Millat Road, Bhaderabad, Karachi, Pakistan; 3 Dr. Fatima Rizvi, MBBS, MPhil, Associate Professor, Department of Pharmacology, Jinnah Medical and Dental College, Shaheed-Millat Road, Bhaderabad, Karachi, Pakistan

**Keywords:** Atorvastatin, Betamethasone Valerate0.1%, Cardio vascular risk, Psoriasis, **PASI:** Psoriasis Area and Severity Index, **DLQI:** Dermatological Life Quality Index, **hsCRP:** Highly sensitive C-reactive protein, **LFTs:** Liver Function Test.

## Abstract

**Objectives:**

To evaluate the effect of Atorvastatin as an adjuvant with betamethasone valerate on disease severity and cardiovascular risks in chronic plaque type psoriatic patients.

**Methods:**

It is an interventional study conducted in Pharmacology Department of BMSI, JPMC with the collaboration of Dermatology Department of JPMC, Karachi. The duration of study was from June 2013 to June 2016. Seventy five psoriatic patients were prescribed Tablet Atorvastatin 40-20 mg/day (40mg for first three months twice daily followed by 20mg once daily for the next three month) plus topical Betamethasone Valerate 0.1% once daily for 6 months (three week apply than one week interval). The efficacy and safety profile of drugs was measured by PASI, DLQI, hsCRP, LFTS and Lipid profile.

**Results:**

The percentage change of PASI is 86.749±0.547, DLQI is 82.697±.2.61 and hsCRP is 40.371±8.505, which showed highly significant improvement in patient at the end of last follow up. LFTs and CPK for safety profile of therapy showed non-significant results.

**Conclusion:**

Atorvastatin used as an adjuvant therapy with currently existing standard therapy (topical betamethasone) in patients having mild to moderate plaque type psoriasis reduces disease severity and cardiovascular risks.

## INTRODUCTION

Topical Corticosteroid remains first line treatment in the management of all grades of psoriasis as a monotherapy and in combination. It acts on cellular level by genomic and non-genomic pathways. It modulates the expression of proinflammatory genes transcription for cytokines, growth factors, adhesion molecules, nitric oxide, prostanoids and autacoids. Topical Betamethasone exerts an anti-inflammatory, antiproliferative and immunosuppressive action.^1^

The side effects of long-term topical Steroid uses are hypopigmentation, skin atrophy, telangiectasiae, dry skin, redness, burning and the risk of microbial growth.^2^ Atorvastatin is a well-known antihyperlipidemic drug and has anti-inflammatory and immuno-modulatory effects.^3^ Statins competitively inhibit 3-hydroxy-3-methylglutaryl coenzyme A (HMG-CoA) reductase which catalyses the rate-limiting step in cholesterol synthesis.^4^

Mevalonate is the precursor not only of cholesterol but also of many non-steroidal-isoprenoid compounds. Which are responsible for the proliferation and migration of smooth muscle cells and causes growth of atherosclerotic plaque and psoriatic plaque. Atorvastatin suppresses intercellular adhesion molecules (ICAM-1) and leukocyte function associated-1 (LFA-1), C-reactive protein (CRP) level, expression of major histocompatibility complex (MHC) class II molecules on macrophages, endothelial cell, smooth muscle cell and nitric oxide generation is another important function of Atorvastatin which involved in psoriasis and cardiovascular risk.^5^ Adverse events are rare that includes myopathy, and abnormal liver enzymes.^6^ The autoimmune diseases and association of atherosclerosis have many similarities. Many common pathways of pathogenesis of inflammation are parallel to each other. The hallmark of psoriasis and cardiovascular risk is an immune mediated inflammatory process. There are many theories that prove that both are related to each other. Thus Statin and Betamethasone modulates this immunoinflammatory mechanism of disease synergistically.^7^,^8^ Few studies have reported that Atorvastatin worsened psoriasis. Based on this study results, it seems that statin represent a promising class of medications used in psoriasis.

Our objective was to evaluate the effect of Atorvastatin as an adjuvant with betamethasone valerate on disease severity and cardiovascular risks in chronic plaque type psoriatic patients.

## METHODS

This interventional study conducted in Pharmacology Department of Basic Medical Science Institute of Jinnah Postgraduate Medical Center with the collaboration of Dermatology Department of JPMC, Karachi. Seventy five psoriatic patients was required for this study, on the basis of a previous study which showed that 75% improvement in PASI score (PASI 75) was achieved in 8 (40%) out of 20 patients receiving Atrovastatin.^9^ In this study 95% confidence interval and absolute precision was 15. By using computer program ‘OpenEpi’, Version. 2. Sixty eight patients complete the study, prescribed tablet Atorvastatin 40 mg twice daily for first three month followed by 20mg once daily for next three month plus Topical Betamethasone Valerate 0.1% once daily for 6 months (three week apply than one week interval). The duration of study was 180 days with six follow up visits from June 2013 to June 2016 including both male and female with age of 25-65 years having PASI scoring of <12 with a hsCRP of ≥3. Those patients were excluded from study which had current statin and steroid therapy in past one month and a history of any other illness, pregnancy and lactation. Informed Consent was taken from all patients and Ethical approval was obtained from Ethical Committee of JPMC.

The current gold standard for assessment of drug efficacy in psoriatic patients is the Psoriasis Area and Severity Index (PASI). A PASI >12 defines severe, PASI 7–12 moderate, and PASI <7 mild psoriasis.^10^,^11^ PASI improvement explained that change in PASI are 50%, 75% and 90%. FDA approved that 50% improvement in PASI means the treatment is efficient clinically.^12^

C-reactive protein is a marker of inflammation that increases in psoriasis. It is used for global index of disease severity. Monitoring CRP level can serve as an important prognostic factor while assessing response to treatment.^13^ Elevated levels of CRP may be an independent risk factor for CVD (cardio vascular diseases) in patients with psoriasis. The plasma levels of CRP in most healthy subjects is usually 1 mg/L.^14^

Quality of life will be evaluated by dermatological quality life index which was assessed on the first day of enrollment and finally reassessed after the therapy. Dermatological life quality index is a useful index for clinical grading of patient‘s quality of life.^15^ Psoriasis is associated with dyslipidemia, a risk factor for cardiovascular diseases. Atorvastatin decreases the S-Triglyceride, S-LDL c and increases S- HDL.^16^

Safety profile of Atorvastatin was measured by Liver function test performed to analyze liver enzymes which are essential in the monitoring of liver diseases.^17^ CPK increases in muscle damage. Atorvastatin rarely induces the myopathy.^18^

### Statically analysis

The data feeding and analysis were through computer software SPSS (Statistical Package of Social Sciences) version 16.0. The results were given in the text analyzed by using repeated measure ANOVA and one way T–test. Only p-values <0.05 was considered significant.

## RESULTS

**Fig.1:**
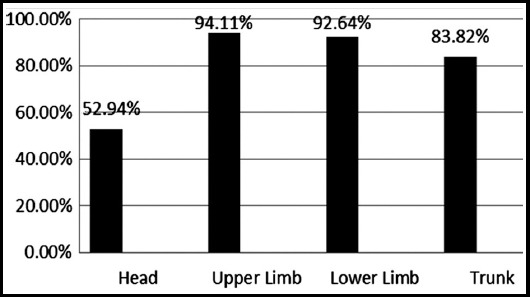
Location of Lesion.

**Fig.2:**
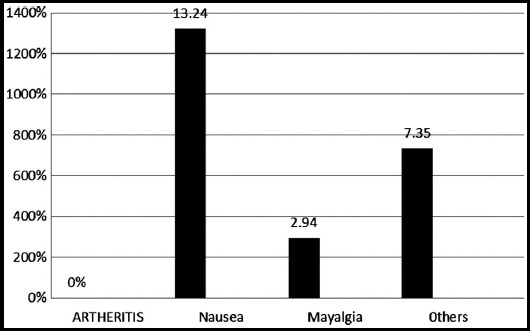
Adverse Events in patients.

The demographic data showed that most of the patient were associated smokers, had positive family history, disturbed sleep with moderate PASI score and the commonest site of lesion was upper limb. Table-I, Fig.1 Combination of drugs showed highly significant change in outcome variables of psoriatic patients at different follow ups visit. There were significant effect on lipid profile of psoriatic patients and no significant effect on safety profile of different doses of Atorvastatin at different intervals Table-II and Table-III. Patients mostly complained of nausea, no one complained of myopathy and other adverse events with higher doses of Atorvastatin plus Betamethasone as shown in Fig.2.

**Table-I:** Demographic Characteristics of Patients.

*Variables*	*Group A*
Age (Years)	47.91 ± 8.42
***Gender***	
Male	52 (76.48)
Female	16 (23.53)
Family History	35 (51.47)
History of Smoking	43 (63.24)
Sleep Disturbed	68 (100))
Duration of Disease	5.57 ± 1.63
Mild-PASI < 7	2 (2.94)
Moderate PASI (7-12)	66 (97.05)

**Table-II:** Psoriasis Area and Severity Index, C- reactive protein & Dermatological Life Quality Index.

*Outcome Variables*	*Baseline*	*Three Month Follow-up*	*Six Month Follow-up*	*P-value*
Psoriasis Area and Severity Index (PASI) (Percentage Change)	10.988±0.711	2.72±0.178 (75.193±0.060)	1.45±0.058 (86.749± 0.547)	0.0001 (0.0001)
CRP (Percentage Change)	3.98 ± 0.33	3.11±0.38 (21.879±6.48)	2.37±0.38 (40.371±8.505)	0.001 (0.0001)
Dermatological Quality Of Life (DLQI) (Percentage Change)	19.77 ± 1.43		3.14±0.53 (82.697±.2.61)	0.001 (0.0001)

**Table-III:** Biochemical Parameters.

*Variables*	*Baseline*	*Third months Follow-up*	*Six Month Follow-up*	*P-value*
Total Cholesterol (mg/dl)	193.5 ± 4.67	183.78 ± 5.17	163.86 ± 5.19	0.001
Triglyceride (mg/dl)	145.79 ± 2.84	139.85 ± 3.26	132.95 ± 3.61	0.001
High Density Lipid (mg/dl)	38.24 ± 1.73	39.07 ± 1.49	40.17 ± 1.44	0.001
Low Density Lipid (mg/dl)	141.62 ± 4.13	135.03 ± 3.99	126.19 ± 4.35	0.001
Alanine Aminotransferase (U/L)	33.544 ± 3.584	33.823 ±3.354	33.941 ± 3.138	0.087
Aspartate Aminotransferase (U/L)	34.544 ± 3.435	34.779 ±3.561	34.720 ± 3.656	0.272
Gamma Glutamyl Transferase (U/L)	37.926 ± 4.042	38.132 ±4.117	38.191 ± 4.197	0.206
Alkaline Phosphatase(U/L)	94.323 ± 6.374	94.455 ±6.374	94.617 ± 6.132	0.299
Total Billirubin (U/L)	0.583 ± 0.105	0.602 ± 0.142	0.611 ± 0.172	0.210
Creatine Phosphokinase (U/L)	79.941 ± 10.52	80.147 ±10.25	80.279 ± 10.03	0.126

## DISCUSSION

We found that the most common sites of lesion were elbows, knees, scalp, umbilicus and lumbar area as dissipated in Fig.1.^19^ All patients reached to PASI-75 at the end of six month. Other studies have reported that tablet Atorvastatin 40 mg improved PASI change upto 75% but their sample size was small. In this study the PASI change in patients showed that the drug was effective and safe.^9^

CRP is the marker of inflammation and cardiovascular risk. The Baseline hsCRP was 3.98±0.33 in this study. The mean percentage change from base line to six month were 40.371±8.505 in patients. The Atorvastatin modulate inflammatory process and reduces LDL–c and CRP by reducing the IL1 and TNFα. In a clinical Miracl study which illustrated that Statin reduced 83% CRP from baseline.^20^

DLQI at Baseline were 19.77 ± 1.43. The greater health problems occur in elderly people and psychological stresses mainly effected in younger ones as observed in this study. The mean percentage change of DLQI at the end of six month were 82.697±.2.61 in patients. Combination of Atorvastatin and Betamethasone Valerate 0.1% gives better results. The Betamethasone is first line treatment in mild to moderate plaque type psoriasis and results are in agreement with Thawornchaisit and Harncharoen who observed that topical Betamethasone gave statistically significant results in reducing PASI score in plaque type psoriasis and they have synergistic effect on hsCRP.^21^

In this study the lipid profile showed proatherogenic values. Therefore psoriasis causes dyslipidemia which is the most common factor of cardiovascular risk. Various external and internal factors lead to proatherogenic lipid profile, these factors such as sedentary lifestyle, genetic influence, stresses, smoking and increase in oxidative injuries which increases morbidity of cardiovascular diseases.^22^ At first follow up of laboratory investigations in third month after the treatment significant difference was found in lipid profile in patients from baseline as shown in Table-III. The mean of percentage change of lipid profile showed that Atorvastatin in high doses produced larger change in lipid profile of patients in this study.^23^,^24^ Corticosteroid also improves inflammation which affects the plasma lipid. Topical corticosteroid have anti-inflammatory, anti-proliferative and immunomodulatory effects.^25^

Other safety profile in this study such as LFTs, and CPK showed no significant change from baseline to third month and six month. CURE-ACS trial has also proved that tablet Atorvastatin in 40 mg as well as 80 mg was safe and well tolerated.^26^

### Limitations of the study

About 63% patients were smokers in this study. Smoking itself related to cardiovascular risk. Stratification as smokers and nonsmokers in results could have been under taken to avoid bias.

## CONCLUSION

This study proved that atorvastatin used as an adjuvant with betamethasone valerate reduces disease severity and cardiovascular risks in plaque type psoriatic patients. It modify the immune function and inhibits inflammatory process thus protects them against cardiovascular risk.
